# Early-Season Avian Deaths from West Nile Virus as Warnings of Human Infection

**DOI:** 10.3201/eid0904.020421

**Published:** 2003-04

**Authors:** Stephen C. Guptill, Kathleen G. Julian, Grant L. Campbell, Susan D. Price, Anthony A. Marfin

**Affiliations:** * U.S. Geological Survey, Reston, Virginia USA; † Hershey Medical Center, Hershey, Pennsylvania, USA; ‡ Centers for Disease Control and Prevention, Atlanta, Georgia

**Keywords:** West Nile virus, surveillance data, early reports, avian deaths, relative risk, dispatch

## Abstract

An analysis of 2001 and 2002 West Nile virus (WNV) surveillance data shows that counties that report WNV-infected dead birds early in the transmission season are more likely to report subsequent WNV disease cases in humans than are counties that do not report early WNV-infected dead birds.

West Nile virus (formal name: *West Nile virus* [WNV]) was first detected in the United States during an encephalitis outbreak in New York City in September 1999 (*1*). Since then, WNV activity has been reported from 42 additional states and the District of Columbia (*2*). Avian, equine, and human illnesses are most often reported. Analysis of surveillance data from 2001 and 2002 chronicles the spread of infection and may provide a means of locating areas where human illness is more likely to occur.

## The Study

Surveillance data have often been used in the study of arboviral disease outbreaks (*3*,*4*). ArboNET, a cooperative WNV surveillance program maintained by the Centers for Disease Control and Prevention and 48 states, five cities, and the District of Columbia, collects surveillance data on a continuous basis. These data include reports of WNV-infected mosquitoes, sentinel animals, dead birds, and ill humans and horses (*5*). In 2001, 328 counties reported a total of 7,333 WNV-infected dead birds (range per county: 1–350). The first WNV-infected dead bird was found on April 8, and the last was found on December 26. Sixty-six human cases of WNV disease were reported from 39 different counties in 10 states,^1^ including two outpatient West Nile fever cases. No single county reported more than four human cases. Onset of illness was on July 13 for the first human case and December 7 for the last reported case. Of particular interest is the date that the first WNV-infected dead bird was found in a given county. These dates ranged from the week ending April 14 to the week ending December 8.

In this retrospective cohort study, all U.S. counties that reported dead WNV-infected birds were categorized on the basis of whether a WNV-infected bird was found early in the transmission season (i.e., before August 5) and whether at least one subsequent human disease case was reported from the county. A relative risk (RR) statistic was calculated as follows: The proportion of counties that reported human cases among the counties that found infected birds before August 5 was divided by the proportion of counties that reported human cases among the counties that did not find infected birds before August 5.

## Results

**Figure 1:**
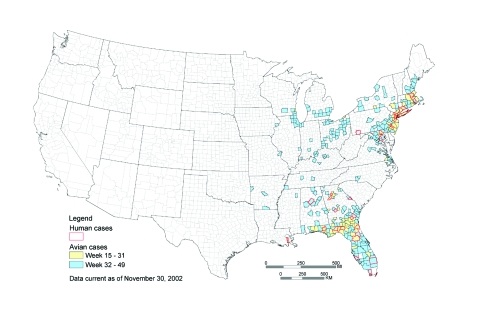
Counties reporting avian deaths and human illness caused by West Nile virus (WNV), January 1–December 31, 2001. Counties reporting human illness are outlined in red. The color within the county indicates the date when the first avian death from WNV was reported in that county. Counties that report dead birds early in the year are more likely to report subsequent disease cases in humans.

Of 93 counties that reported at least one WNV-infected bird before August 5, 28 (30%) subsequently reported a human WNV disease case in 2001 compared to 11 (4.7%) of 235 counties that did not report an infected bird (RR 6.43, 95% confidence interval [CI] 3.34 to 12.38). In other words, in 2001, counties that reported a WNV-infected dead bird before August 5 were more than six times more likely than other counties to report a human WNV disease case (Figure 1).

As Figure 1 shows, the 2001 outbreak had two distinct geographic foci, the Northeast and the Southeast United States. These areas were analyzed separately to determine if the correlation between WNV-positive birds and human cases was true in different ecologic regions. The Northeast region consisted of Maryland, New Jersey, Pennsylvania, New York, Connecticut, Rhode Island, and Massachusetts. The Southeast region consisted of Florida, Georgia, Alabama, and Louisiana. The Northeast region contained 22 of the 39 counties in which human cases occurred. The Southeast contained the remaining 17 counties. RR statistics were significant for both regions (Northeast: RR 11.57, 95% CI 3.58 to 47.99; Southeast: RR 2.38, 95% CI 0.89 to 6.39).

**Figure 2:**
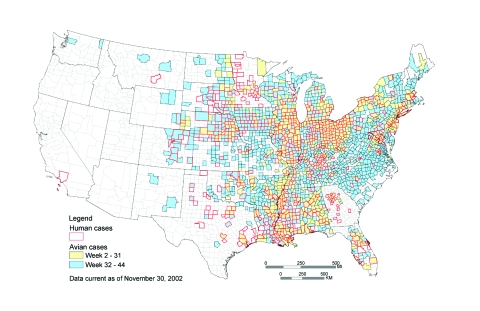
Counties reporting avian deaths and human meningitis/encephalitis caused by West Nile virus (WNV), January 1–November 30, 2002. Counties reporting human illness are outlined in red. The color within the county indicates the date when the first avian death from WNV was reported in that county. Counties that report dead birds early in the year are more likely to report subsequent disease cases in humans

Recently, provisional totals for the 2002 WNV surveillance data have become available through ArboNET. Given the great increase in the geographic extent and the 50-fold increase in the number of human cases of WNV, we repeated this analysis by using the provisional data for 2002 to see if similar results would be obtained. A great deal of variation in the reporting of WNV fever cases has occurred between states. For this reason, only WNV meningitis and encephalitis cases were included in this analysis. In the provisional figures for 2002, a total of 504 counties reported human cases of WNV meningoencephalitis, and 1,719 counties reported WNV-infected birds. Of 632 counties that reported at least one WNV-infected bird before August 4, a total of 284 (45%) subsequently reported a human WNV disease in 2002 compared to 220 (19%) of 1,162 counties that did not report an infected bird (RR 2.37, 95% CI 2.05 to 2.75). Thus in 2002, counties that reported a WNV-infected dead bird before August 4 were more than two times more likely than other counties to report a human case of WNV disease (Figure 2).

The early August date (end of the 31st week of the year) used for classifying the surveillance data was selected by empirical analysis of the WNV epidemiologic curves. This date approximates the inflection point where the rapid increase in case reports occurs. Using an earlier date for classifying the cases results in an increased value for the RR statistic but a decrease in sensitivity.

This type of analysis could possibly be refined by stratifying surveillance data by the number of birds and humans tested to compensate for variations in the intensity of the surveillance effort. Factors such as the size of the human population also may affect the number of dead birds sighted and the number of persons exposed to WNV-infected mosquitoes. Other researchers have attempted to address these issues (*6*–*8*). In addition, we are analyzing the data to determine if the risk for human illness is greater the earlier the positive bird (or other indication of epizootic transmission) is detected.

However, the aim of this study was to see if a simple analysis of surveillance data could provide useful indicators of human disease risk. The results of our analysis suggest that, in counties where an avian epizootic is present early in the transmission season, subsequent WNV disease in humans is more likely. An early epizootic may indicate viral activity that has sufficient time to escalate to high levels before the end of the transmission season. WNV-infected dead birds found in spring or early summer thus may be a warning for increased human risk for WN viral disease.

## Footnotes

1 These figures do not include a human case in New York that was not located to the county level.
